# Effect of mushrooms on obesity in animal models: study protocol for a systematic review and meta-analysis

**DOI:** 10.1186/s13643-019-1205-3

**Published:** 2019-11-26

**Authors:** Denise Grotto, Isabella Ferreira Camargo, Katia Kodaira, Lauren Giustti Mazzei, Juliana Castro, Raquel Andrade Leite Vieira, Cristiane de Cásia Bergamaschi, Luciane Cruz Lopes

**Affiliations:** Pharmaceutical Sciences Graduate Course, University of Sorocaba, Rodovia Raposo Tavares, Km 92.5, Sorocaba, SP 18023-000 Brazil

**Keywords:** Obesity, Mushroom, Body weight loss, Animal experimentation, Study protocol, Systematic review

## Abstract

**Background:**

Obesity and its consequences are worldwide epidemic problem; therefore, studies with strategies and mechanisms that favor weight loss to improve outcomes in health are necessary. Effects of mushrooms on body weight are uncertain. The aim of this systematic review is to determine the efficacy of mushrooms in weight loss in animal preclinical models.

**Method:**

This is a systematic review of preclinical studies of animal models of obesity (any type of non-aquatic mammal), which were exposed to edible and medicinal mushrooms orally in comparison with the control. The following databases will be used: MEDLINE (PubMed), Web of Science, BIOSIS, SCOPUS, and gray literature. There will be no restriction of language, date, or publication status. The primary outcome will be body weight loss. And the secondary outcomes include the total amount of food consumed by the animals, analysis of metabolic parameters, inflammatory mediators, mortality for any causes, and any adverse effect reported. A team of reviewers will select, in pairs and independently, the titles and abstracts, extract data from qualifying studies, and assess bias risk (using SYstematic Review Centre for Laboratory animal Experimentation SYRCLE’s risk of bias tool and the Collaborative Approach to Meta-Analysis and Review of Animal Data from Experimental Studies (CAMARADES) checklist). The standardized mean difference (SMD) will be calculated to measure treatment effect, with 95% confidence intervals (95% CI). The heterogeneity between-study will be calculated by *I*^2^ inconsistency values and Cochran’s *Q* statistical test, where *I*^2^ > 50% and/or *p* < 0.10 suggest high heterogeneity meta-analyses of random effects will be conducted as possible.

**Discussion:**

Although many experimental studies about the effects of mushrooms on obesity have already been published, there is still no consensus in the literature. This study will provide evidences of preclinical research on mushrooms and their relation to body weight loss in animal models of obesity, being non-aquatic mammals. Also, this systematic review will show the limitations and strengths of the studies available in the literature, as well as it will to encourage the financing of new studies by public health managers and governmental entities.

**Systematic review registration:**

PROSPERO (CRD42019125299).

## Background

Obesity is defined as excessive accumulation of fat in adipose tissue; a consequence of imbalance involving intake, energy expenditure, and physical activity patterns [1, 2]. Drastic changes in social and eating habits have significantly impacted in health and nutritional status of individuals [3, 4].

The World Health Organization (WHO) considers obesity as one of the most obvious and neglected public health problems that threaten the present days [1]. The prevalence of obesity in the world population shows us a significant and accelerated growth of the disease in the last decades. Obesity has become a global epidemic, affecting not only developed but also developing countries and among all segments of society [5].

Besides being considered as a chronic disease, obesity is an important risk factor to no communicable chronic diseases such as diabetes, cardiovascular diseases, cancer, apnea, and osteoarthritis. The health consequences are numerous, such as the risk of premature death, debilitating complaints that affect the quality of life, and psychosocial disorders [1, 6, 7].

Obesity is increasingly considered a priority for human health and its treatment is essential due to its devastating effect on health as well as problems related to comorbidities [8]. Lifestyle changes are the first choice to start treating obesity. Anti-obesity medications should be started when the behavioral change fails. Adverse effects of medication and potential for drug abuse are the main limitations to the indication of this therapy. Therefore, the search for alternative therapies for the treatment of obesity has increased considerably in the scientific environment [8–10].

Mushrooms have been used as foodstuff and folk medicine for thousands of years because of their nutritional and medical properties. Edible mushrooms, especially in the order Agaricales, have low calorie, low fat concentration, and high protein and fiber content, and they have the essential amino acids [11, 12]. Besides being a source of vitamins and minerals, edible mushrooms also have several biologically functional compounds, such as polysaccharides, glycoproteins, and antioxidants, which have been used mainly as antitumor and immunostimulant [11, 13, 14]. Likewise, medicinal mushrooms also have different functional compounds, with similar activities than edible mushrooms; antibacterial, hepatoprotective, anti-inflammatory, antitumor, and antioxidant action [15]. However, many studies have reported other beneficial from these mushrooms, as the anti-obesity, anti-diabetic, and anti-hyperlipidemic effects [16–22].

Iñiguez and colleagues reported that supplementation with *Agaricus bisporus* prevented excessive body weight gain and liver steatosis induced by high-fat-diet feeding. In the same way, dietary *Sparassis crispa* exhibited anti-obesity effect in rats with diet-induced obesity [17]. On the other hand, oral administration *Lentinula edodes* for 30 days was not sufficient to reduce body weight in a high-fat-diet feeding group [18]. Thus, although edible and medicinal mushrooms have been shown anti-obesity effects in numerous preclinical studies, the results are far from conclusive.

To our knowledge, no systematic review or meta-analysis has been reported critical evidence regarding the effects of mushrooms in animal models of obesity. Therefore, a systemic review and, if possible, a meta-analysis is proposed in order to assess the anti-obesity effects of edible and medicinal mushrooms in animal models of obesity.

### Research question

What are the effects of edible and medicine mushrooms on the body weight loss in animal models of obesity?

## Methods/design

This protocol was delineated according to the recommendations from the Preferred Reporting Items for Systematic reviews and Meta-Analyses for Protocols (PRISMA-P) [23] and the recommendations for reporting of systematic reviews and meta-analyses of animal experiments [24, 25]. The protocol was registered on International Prospective Register of Systematic Reviews (PROSPERO—CRD42019125299) (https://www.crd.york.ac.uk/prospero/display_record.php?RecordID=125299).

### Eligibility criteria

#### Types of studies

The systematic review will include controlled studies (randomized and non-randomized), which evaluated the effect of edible and medicinal mushrooms on preclinical animal models of obesity. Both unpublished and published studies are eligible for inclusion. There is no restriction of language, date, or publication status.

#### Types of animal models

We will include studies that used any type of non-aquatic mammals’ model, which have developed obesity genetically, physiological, through dietary, surgical, or seasonal obesity. Each of these models mimics at least part of the various pathophysiological aspects of obesity.

#### Types of comparators

The comparison group will include animals whose obesity was preclinically induced but have not undergone any intervention.

#### Types of intervention

The intervention group will include animals that received edible or medicinal mushrooms to investigate body weight loss. Although there are different types of mushrooms, only studies with edible or medicinal mushrooms will be accepted. Only oral administrations of mushrooms will be included. We will select studies that have used the body of the mushroom (fruit body), powder formulations, or extracts of the mushrooms. To be included in our analysis, mushrooms must have been administered during or after the induction of experimental obesity.

### Exclusion criteria

For this systematic review, *in vitro* experiments, case report studies, cohort studies, abstracts of congress, letters to the editor, and all human studies will be excluded. Studies comparing animal models of obesity with healthy animals and mushroom administration by other routes, such as intravenous, intramuscular, dermal, intradermal, and intraperitoneal routes, will also be excluded.

Exclusion criteria still comprise studies using edible or medicinal mushrooms as pre-treatment, studies using substances isolated from mushrooms and co-intervention studies, because of the risk of contamination. Moreover, hallucinogenic, poisonous, and toxic mushrooms will not be included in the systematic review.

### Types of outcome measures

#### Primary

Primary outcome measures will be loss in body weight, assessed through body weight, and measured at the highest follow-up time following administration of the intervention.

#### Secondary

Secondary outcomes include the total amount of food consumed by the animals; analysis of metabolic parameters, such as total cholesterol, high-density lipoprotein, low-density lipoprotein, triglycerides, glycaemia, inflammatory mediators (interleukin-1 and tumor necrosis factor), mortality for any causes, and any adverse event reported by the authors in any time after mushroom administration.

### Search methods for identification of studies

#### Electronic searches

The following electronic databases will be used: MEDLINE (PubMed); Web of Science, BIOSIS, SCOPUS, Google Scholar from its inception until November 2019. There will be no restriction of language, publication date, or publication status. We will also use Search Filter for laboratory animals, restricting for in vivo studies.

#### Searching other resources

Gray literature and manual search will be included in search criteria. Gray literature compiles materials and researches that are not covered in the databases mentioned, as well as the sites of animal research organizations and Google Scholar. In manual search, two reviewers (IC and KK) will check the reference list or citations found in secondary studies to verify and identify possible eligible studies. Whenever necessary, the authors of the main studies will be contacted for additional information.

#### Search strategy

The main terms “Mushroom,” “Obesity,” “Body Weight Loss,” and “Animal Experimentation,” indexed in the MeSH system, will be combined. First, the terms and their synonyms, separately, will be searched. Then, a second research will be done combining and crossing the terms. The Research Filter for laboratory animals [26] will also be used, restricting it to in vivo studies with non-aquatic mammals. Details of the PubMed search strategy appear in Additional file 1.

## Data collection and analysis

### Selection of studies

Pairs of reviewers (DG and KK, IFC and JC, RALV and CCB), independently, will screen titles and abstracts. Duplicates will be removed during the screening. Disagreements between researchers will be resolved by consensus or third review (LCL). After, same reviewers, in pairs and independently, will evaluate the full text of studies using a standardized form with included and excluded criteria. In case of duplicate publication, we will use the article with more complete data.

### Data extraction

The same reviewers, working in pairs, will independently extract the data and will record information: (i) study design (number and type of studies—controlled trials randomized or non-randomized, unpublished and published studies), (ii) study characteristics (author, year of publication, study title), (iii) characteristics of the included animals and animal model (animal species, obesity model, age, gender, husbandry conditions, number of animals in intervention, and comparator group), (iv) interventions (animals that received edible or medicinal mushrooms, time and description of preparation, route given), (v) outcomes of interest (body weight, total amount food consumed, metabolic parameters and inflammatory mediators, death, adverse event). Before starting data abstraction, we will conduct calibration exercises to ensure consistency between reviewers.

### Assessment of risk of bias and quality assessment in included studies

Methodological quality of the included studies will be assessed according to the SYstematic Review Centre for Laboratory animal Experimentation SYRCLE’s risk of bias tool [27] and the Collaborative Approach to Meta-Analysis and Review of Animal Data from Experimental Studies (CAMARADES) checklist that evaluate the following: publication in a peer-reviewed journal, statement of control of temperature, randomized treatment allocation, blinded assessment of outcome, reporting of blinding of the operator, appropriate animal model, reporting of a sample size calculation, compliance with animal welfare regulations, statement of potential conflict of interest, and complete follow-up [28, 29].

### Measures of treatment effect

Any type of continuous or dichotomous data will be collected. We will calculate standardized mean difference (SMD), odds ratio (OR), and related 95% confidence interval (95% CI) for each outcome using Cohen’s method to normalize the different animal species.

Dichotomous data will be calculated as risk ratio (RR) with 95% CI.

### Assessment of heterogeneity and data synthesis

To determine whether the included studies have enough homogeneity for meta-analyses, we will estimate between-study heterogeneity by calculating *I*^2^ inconsistency values and Cochran’s *Q* statistical test, where *I*^2^ > 50% and/or *p* < 0.10 suggest high heterogeneity. Heterogeneity will be defined according to the *I*^2^ range: 0 to 40% indicated no important heterogeneity, 40 to 60% moderate heterogeneity, 60 to 90% substantial heterogeneity, and > 90% indicating considerable heterogeneity [30].

The meta-analyses of random effects will be conducted, by each outcome, when there are at least two studies. Data analysis will be performed by STATA® Statistical software version 14.2 (Stata Corp, College Station, USA). If quantitative synthesis is not appropriate, we will construct summary tables and provide a narrative synthesis.

### Grading the quality of evidence

GRADE method will be used to interpret the results [31, 32]. The quality of evidence of the studies will be graded at four levels: very low, low, moderate, and high, based on risk of bias, inconsistency, indirectness, imprecision, and publication bias. Quality ratings will be made separately for each outcome. Based on study limitations, authors will make an overall judgment whether the quality of evidence for an outcome warrants downgrading. As GRADE’s indirectness domain is only applicable to human subjects, we will downgrade the quality of evidence by 1 (as serious indirectness). Also, the quality rating will be downgrade to level 1 if the evidence is classified as “serious” and to two levels if it is classified as “very serious.” However, it potential limitations will not be likely to lower confidence in the effect estimate, the evidence will not be downgraded.

### Assessment of publication bias

A graphical funnel plot will be used to investigate (at least 10 studies contributed to a pooled analysis), whether publication bias will be present in the studies included in the review [33].

## Discussion

This systematic review and meta-analysis aim to provide and inform researchers and practitioners in the corresponding areas (clinicians and health regulators) of the evidence on preclinical research and relevant existing evidence regarding mushrooms and their relationship with body weight loss in animal models of obesity, being non-aquatic mammals. Also, this study aims to present the strengths and limitations of the studies available in the literature and offer future perspectives in this field.

Although many experimental studies on the effects of mushrooms on obesity have already been published, there is still no consensus in the literature. Therefore, a systematic analysis of existing experimental studies is necessary.

## Supplementary information

**Additional file 1.** Search Strategy for database.

## Data Availability

Not applicable.

## Abbreviations

95% CI: 95% confidence intervals
CAMARADES: Collaborative Approach to Meta-Analysis and Review of Animal Data from Experimental Studies
MeSH: Medical Subject Headings
OR: Odds ratio
PRISMA-P: Preferred Reporting Items for Systematic reviews and Meta-Analyses for Protocols
PROSPERO: International Prospective Register of Systematic Reviews
RR: Risk ratio
SMD: Standardized mean difference
SYRCLE: SYstematic Review Center for Laboratory animal Experimentation
WHO: World Health Organization
