# Supplementary material for: Effect of mushrooms on obesity in animal models: study protocol for a systematic review and meta-analysis
Source: Syst Rev. 2019 Nov 26;8:288. doi: 10.1186/s13643-019-1205-3 (PMC6878714; doi:10.1186/s13643-019-1205-3)
Supplement: Supplementary file 1 — Additional file 1. Search Strategy for database. [file 13643_2019_1205_MOESM1_ESM.docx]

**Additional file 1:** Search Strategy for database.

| **Search Strategy**:  " Mushroom" AND " Obesity" AND “Body Weight Loss” AND "Animal experimentation"  **Data base: MEDLINE via PubMed <1946 to November 2018>**  **Search Strategy:**   1. Agaricales 2. Agaricale 3. Mushrooms 4. Mushroom 5. Agaricaceae 6. Shiitake Mushrooms 7. Mushroom, Shiitake 8. Mushrooms, Shiitake 9. Shiitake Mushroom 10. Lentinula edodes 11. Lentinula edode 12. edodes, Lentinula 13. Lentinus edodes 14. Lentinus edode 15. edode, Lentinus 16. Pleurotus 17. Oyster Mushrooms 18. Mushroom, Oyster 19. Mushrooms, Oyster 20. Oyster Mushroom 21. Pleurotus ostreatus 22. Agaricus 23. Lentinula 24. Lentinulas 25. Lentinus 26. (((((((((((((((((((((((((Agaricales) OR Agaricale) OR Mushrooms) OR Mushroom) OR Agaricaceae) OR Shiitake Mushrooms) OR Mushroom, Shiitake) OR Mushrooms, Shiitake) OR Shiitake Mushroom) OR Lentinula edodes) OR Lentinula edode) OR edodes, Lentinula) OR Lentinus edodes) OR Lentinus edode) OR edode, Lentinus) OR Pleurotus) OR Oyster Mushrooms) OR Mushroom, Oyster) OR Mushrooms, Oyster) OR Oyster Mushroom) OR Pleurotus ostreatus) OR Agaricus) OR Lentinula) OR Lentinulas) OR Lentinus) 27. Obesity 28. Body weight 29. Body Weights 30. Weight, Body 31. Weights, Body 32. (((((Obesity) OR Body weight) OR Body Weights) OR Weights, Body) OR Weight, Body) 33. Weight Loss 34. Loss, Weight 35. Losses, Weight 36. Weight Losses 37. Weight Reduction 38. Reduction, Weight 39. Reductions, Weight 40. Weight Reductions 41. Diet, Reducing 42. Diets, Reducing 43. Reducing Diet 44. Reducing Diets 45. Weight Reduction Diet 46. Diet, Weight Reduction 47. Diets, Weight Reduction 48. Weight Reduction Diets 49. Weight Loss Diet 50. Diet, Weight Loss 51. Diets, Weight Loss 52. Weight Loss Diets 53. Body Weight Maintenance 54. Body Weight Maintenances 55. Maintenance, Body Weight 56. Maintenances, Body Weight 57. Weight Maintenance, Body 58. Weight Maintenances, Body 59. Weight Gain 60. Gain, Weight 61. Gains, Weight 62. Weight Gains 63. ((((((((((((((((((((((((((((((((((((Weight Loss) OR Loss, Weight) OR Losses, Weight) OR Weight Losses) OR Weight Reduction) OR Reduction, Weight) OR Reductions, Weight) OR Weight Reductions) OR Diet, Reducing) OR Diets, Reducing) OR Reducing Diet) OR Reducing Diets) OR Weight Reduction Diet) OR Diet, Weight Reduction) OR Diets, Weight Reduction) OR Weight Reduction Diets) OR Weight Loss Diet) OR Diet, Weight Loss) OR Diets, Weight Loss) OR Weight Loss Diets) OR Body Weight Maintenance) OR Body Weight Maintenances) OR Maintenance, Body Weight) OR Maintenances, Body Weight) OR Weight Maintenance, Body) OR Weight Maintenances, Body) OR Weight Gain) OR Gain, Weight) OR Gains, Weight) OR Weight Gains) 64. animal experimentation 65. models animal 66. Animals 67. animal population groups 68. vertebrates 69. mammals 70. primates 71. Artiodactyla 72. carnivora 73. chiroptera 74. elephants 75. insectivora 76. lagomorpha 77. marsupialia 78. perissodactyla 79. rodentia 80. Xenarthra 81. haplorhini 82. platyrrhini 83. catarrhini 84. cercopithecidae 85. hominidae 86. gorilla gorilla 87. pan troglodytes 88. pongo pygmaeus 89. pongo pygmaeus pygmaeus 90. animal 91. mice 92. mus 93. mouse 94. murine 95. rats 96. rat 97. murinae 98. muridae 99. hamster 100. hamsters 101. cricetinae 102. rodentia 103. rodent 104. rodents 105. pigs 106. pig 107. swine 108. swines 109. piglets 110. piglet 111. boars 112. boar 113. sus scrofa 114. ferrets 115. ferret 116. polecat 117. mustelaputorius.mp. 118. guineapig 119. guineapigs 120. cavia 121. callithrix 122. marmosets 123. marmoset 124. cebuella 125. hapale 126. octodon 127. chinchilla 128. chinchillas 129. gerbillinae 130. gerbil 131. gerbils 132. jirds 133. jird 134. merione 135. meriones 136. rabbit 137. rabbit’s 138. hares 139. hare 140. hare’s 141. cats 142. cat 143. carus 144. felis 145. dogs 146. dog 147. canine 148. canines 149. canis 150. sheep 151. sheeps 152. mouflon 153. mouflons 154. ovis 155. goats 156. goat 157. capra 158. capras 159. haplorhini 160. monkey 161. monkeys 162. anthropoidea 163. anthropoids 164. saguinus 165. tamarin 166. leontopithecus 167. tamarins 168. hominidae 169. ape 170. apes 171. pan 172. paniscus 173. troglodytes 174. gibbon 175. gibbons 176. chimpanzee 177. chimpanzees 178. chimpanze 179. bush baby 180. prosimios 181. bush babies 182. galagos 183. galago 184. pongidae 185. gorilla 186. gorillas 187. pongo 188. pygmaeus 189. pongo pygmaeus 190. orangutans 191. lemur 192. lemurs 193. horse 194. horses 195. equus 196. cow 197. calf 198. bull 199. sciuridae 200. squirrel 201. squirrels 202. chipmunk 203. chipmunks 204. suslik 205. susliks 206. vole 207. voles 208. lemming 209. lemmings 210. muskrat 211. muskrats 212. lemmus 213. otter 214. otters 215. marten 216. martens 217. martes 218. weasel 219. weasels 220. badger 221. badgers 222. mink 223. minks 224. sable 225. sables 226. gulo 227. gulos 228. wolverine 229. wolverines 230. mustela 231. alpaca 232. alpacas 233. camelid 234. camelids 235. guanaco 236. guanacos 237. chiroptera 238. chiropteras 239. bat 240. fox 241. foxes 242. donkey 243. donkeys 244. mule 245. mules 246. zebra 247. zebras 248. shrew 249. shrews 250. bison 251. bisons 252. buffalo 253. buffaloes 254. deer 255. deers 256. bear 257. bears 258. panda 259. pandas 260. fitch 261. beaver 262. beavers 263. jerboa 264. jerboas 265. capybara 266. capybaras 267. ((((((((((((((((((((((((((((((((((((((((((((((((((((((((((((((((((((((((((((((((((((((((((((((((((((((((((((((((((((((((((((((((((((((((((((((((((((((((((((((((((((((((((((((((((((((((((((((((((((((((((animal experimentation) OR models animal) or animals) OR animal population groups) OR vertebrates) OR mammals) OR primates) OR artiodactyla) OR carnivora) OR chiroptera) OR elephants) OR insectivora) OR lagomorpha) OR marsupialia) OR perissodactyla) OR rodentia) OR xenarthra) OR haplorhini) OR platyrrhini) OR catarrhini) OR cercopithecidae) OR hominidae) OR gorilla gorilla) OR pan troglodytes) OR pongo pygmaeus) OR pongo pygmaeus pygmaeus) OR animal) OR mice) OR mus) OR mouse) OR murine) OR rats) OR rat) OR murinae) OR muridae) OR hamster) OR hamsters) OR cricetinae) OR rodentia) OR rodent) OR rodents) OR pigs) OR pig) OR swine) OR swines) OR piglets) OR piglet) OR boar) OR boars) OR sus scrofa) OR ferrets) OR ferret) OR polecat) OR guineapigs) OR guineapig) OR cavia) OR callithrix) OR marmoset) OR marmosets) OR cebuella) OR hapale) OR octodon) OR chinchilla) o OR r chinchillas) OR gerbillinae) OR gerbil) OR gerbils) OR jird) OR jirds) OR merione) OR meriones) OR rabbit) OR rabbit’s) OR hares) OR hare) OR hare’s) OR cats) OR cat) OR carus) OR felis) OR dog) OR dogs) OR canine) OR canines) OR canis) OR sheep) OR sheeps) OR mouflon) OR mouflons) OR ovis) OR goats) OR goat) OR capra) OR capras) OR haplorhini) OR monkey) OR monkeys) OR anthropoidea) OR anthropoids) OR saguinus) OR tamarin) OR leontopithecus) OR tamarins) OR hominidae) OR ape) OR apes) or pan) OR paniscus) OR troglodytes) OR gibbon) OR gibbons) OR chimpanzee) OR chimpanzees) OR chimpanzes) OR bush baby) OR prosimios) OR bush babies) OR galagos) OR galago) OR pongidae) OR gorilla) OR gorillas) OR pongo) OR pygmaeus) OR pongo pygmaeus) OR orangutans) OR lemur) OR lemurs) OR horse) OR horses) OR equus) OR cow) OR calf) OR bull) OR sciuridae) OR squirrel) OR squirrels) OR chipmunk) OR chipmunks) OR suslik) OR susliks) OR vole) OR voles) OR lemming) OR lemmings) OR muskrat) OR muskrats) OR lemmus) OR otter) OR otters) OR marten) OR martens) OR martes) OR weasel) OR weasels) OR badger) OR badgers) OR mink) OR minks) OR sable) OR sables) OR gulo) OR gulos) OR wolverine) OR wolverines) OR mustela) OR alpaca) OR alpacas) OR camelid) OR camelids) OR guanaco) OR guanacos) OR chiroptera) OR chiropteras) OR bat) OR fox) OR foxes) OR donkey) OR donkeys) OR mule) OR mules) OR zebra) OR zebras) OR shrew) OR shrews) OR bison) OR bisons) OR buffalo) OR buffaloes) OR deer) OR deers) OR bear) OR bears) OR panda) OR pandas) OR fitch) OR beaver) OR beavers) OR jerboa) OR jerboas) OR capybara) OR capybaras) 268. 26 AND 32 AND 69 AND 273 |
| --- |
